# Bioethics curriculum for undergraduate medical students: an evaluation study utilizing mixed methods approach

**DOI:** 10.1186/s12909-024-05376-4

**Published:** 2024-04-08

**Authors:** Anita Anis Allana, Syeda Kauser Ali, Kulsoom Ghias

**Affiliations:** 1 Department for Educational Development, Aga Khan University, Stadium Road, P.O. Box 3500, Karachi, 74800 Pakistan; 2 Institute of Medical Education, Jinnah Sindh Medical University, Rafiqui H. J. Iqbal Shaheed Road, Karachi, 75510 Pakistan; 3 Department of Biological and Biomedical Sciences, Aga Khan University, Stadium Road, P.O. Box 3500, Karachi, 74800 Pakistan

**Keywords:** Evaluation, Curriculum, Ethics, Medical education research

## Abstract

**Background:**

The undergraduate bioethics curriculum introduced in a private medical college in Pakistan in 1988 and revised in 2009 has evolved over time to incorporate globally relevant innovations, including integration of bioethics spirally within an existing problem-based learning curricular framework. The present evaluation study shares the results of this integrated bioethics curriculum delivered for 10 years across the five-year undergraduate medical curriculum. The study assessed the effectiveness of the curriculum in terms of student achievement, appropriateness of course contents and efficiency of instructional methods.

**Methods:**

The study utilized a mixed method sequential explanatory design. The quantitative method was used in the first phase to gather data by utilizing a structured online questionnaire. This was followed by the second phase of qualitative methods to explain the findings of the first phase and enrich the data gathered. This phase was based on focus group discussions and document review.

**Results:**

Student and faculty responses showed the curriculum contents to be relevant, informative, and appropriate as per learning objectives and student achievement. Multi-modal instructional methods used were stated to be effective and engaging; small group teaching and shorter sessions suggested to be preferable for fostering discussion and maintaining student engagement and attention. Large class formats were stated to be less effective. Students affirmed the contribution of bioethics education to their personal and professional development and ethical positioning. The majority of students agreed that the curriculum contributed to their knowledge acquisition (60.3—71.2%), skill development (59.41—60.30%) and demonstration of ethical/professional behavior (62.54—67.65%). The ranges indicate agreement with related sets of questions.

Participants suggested that the curriculum could be further strengthened by better integration in clinical years, role modelling and providing opportunities for application in clinical health care settings. Moreover, topics like ethical issues related to the use of social media, public health ethics and ethics and law were suggested as additions to the existing curriculum. These findings have regional and global relevance for the development and assessment of effective bioethics curricula.

**Conclusion:**

An effective bioethics curriculum for undergraduate medical education should run longitudinally across the 5 year curriculum and be integrated in the modules and clerkships. Basic acquisition of knowledge and skills takes place in Years 1 & 2 with reinforcement and application in Years 3–5. Learning embedded in an integrated curriculum can help students recognize, critically analyze and address ethical dilemmas. Involvement and commitment of the clinical faculty is essential for reinforcing the ethical principles and concepts learnt in the earlier years.

## Background

Driven by historical lessons garnered from tragic events earlier in the century, bioethics was formally recognized as a discipline in the second half of the twentieth century [1]. Medical schools in the United States and United Kingdom were early adopters of bioethics teaching, guided by rapid developments in the fields of science, medicine and technology, prevailing rights movements and other pivotal societal changes. Bioethics curricula were incorporated across programmatic levels, from undergraduate to postgraduate and in continuing professional education [2–4]. Other parts of the world, including the Middle East, South Asia and the Asia–Pacific region, soon followed. Bioethics education introduced in medical curricula largely in the 1990s was less structured and often inconsistent [5]. In many countries in Africa and Asia, medical colleges still do not have formal integrated bioethics curriculum or defined training standards [6–9]. Many bioethics training programs that have been implemented in low- and middle-income countries (LMICs) have been prompted by the need to build capacity for ethical provision of healthcare and conduct of research [10, 11]. These curricula focus on contextual relevance by bridging the theory-to-practice divide and engage learners through interactive pedagogy, relevant technology, and integrated curricular encounters [12].

Bioethics education and training has evolved with increased recognition of the need for contextually relevant rather than transplanted curricula [10, 13]. The dichotomy in the purpose of bioethics education, seen by some as a means of creating virtuous physician; and by others as a means of providing physicians with a skill set for analyzing and resolving ethical dilemmas, has made it difficult to agree upon the curriculum design, delivery and assessment methods [2]. Evaluation studies of the bioethics curricula have offered useful lessons, for example on the effectiveness of small group learning [4], near-peer teaching approaches [14] and impact of online bioethics courses [10]. For most bioethics curricula implemented and evaluated in the global South, results have been limited to a subset of students and rarely extended to include feedback from other stakeholders such as faculty [12, 15, 16]. Studies conducted in the region have stressed on the relevance of bioethics education for medical students [17, 18], and emphasized the importance of cultural context and its influence on the ethics curriculum [19].

To the best of our knowledge no study whether in developing or developed countries has evaluated an integrated bioethics curriculum that spans across the entire 5 years of medical school and has been delivered, tested and improved continuously for 10 years. Also mixed methods approach involving students and faculty has hardly been used for evaluation of bioethics curricula.

Here we present a study designed to evaluate a bioethics curriculum integrated in a five-year undergraduate medical education program that has been delivered for over ten years at a private medical college in Pakistan. This revised curriculum implemented in the university since 2009 has evolved over time to incorporate global innovations in teaching medical students within an existing curricular framework using problem based learning (PBL) to facilitate contextual understanding and application of knowledge [20–22]. Bioethics teaching was introduced in the medical curriculum at this private medical college in Karachi, Pakistan in 1988 to develop graduates who are well-informed in the history and philosophy of ethics and bioethics and are ethical in their thinking and practice. As detailed in an earlier publication, the curriculum was revisited and revised in 2009 by a multidisciplinary voluntary group [23]. The revised curriculum spans all five years of the spiral undergraduate medical education program and is integrated within the existing curriculum in respective system-based modules/clinical clerkships. The curriculum covers various ethical issues using real-life cases and contextually relevant scenarios [23]. In the first two years, the content is a mixture of moral philosophy and applied clinical ethics. Lectures, discussions, brainstorming, problem solving exercises, videos/movies, case studies among others, are used to support the more formal teaching sessions. Topics include reasoning for moral dilemmas through reflexivity and logic, principles of bioethics, major ethical theories, history of bioethics, problematization of bioethics, the role of the doctor, consent and confidentiality, truth-telling, ethics of behavior, the ethics of organ trade, ethical issues related to reproductive health, conflict of interest, priority setting and rights of patients and vulnerable groups. These topics are integrated within the system-based modules. In subsequent years, bioethics is integrated within clerkships and also offered as workshops along with topics like gender, culture and communication. The ultimate goal of the curriculum is to facilitate acquisition of relevant knowledge, skills and attitudes necessary for a medical graduate to appreciate and critically process ethical dilemmas, to adhere to the highest standards of professional behavior behaving ethically in caring for patients and in interactions with patients’ families and others involved in patient care.

As a part of continuous quality improvement process, a formative evaluation was conducted to determine the effectiveness of the bioethics curriculum, specifically with respect to course contents, instructional methods, and student achievement. A review of bioethics educational programs stressed that it is important to evaluate the curriculum to determine not only appropriate teaching and assessment methods but also identify the staff and faculty development needs [2].

This study, irrespective of the geographical context, evaluates and shares lessons from an integrated bioethics curriculum that spans across an entire five-year undergraduate medical curriculum and has been delivered, evaluated and improved continuously for over a decade. It is also important in the local context as regulatory bodies have mandated bioethics education in the undergraduate curriculum in medicine and dentistry. The findings of this study may provide guidance to the developers and implementers of the bioethics curriculum not only in Pakistan, but also in the region.

## Methods

This evaluation study aimed to assess the effectiveness of the bioethics curriculum in terms of student achievement, appropriateness of course contents and efficiency of instructional methods using a mixed method sequential explanatory design [24].

In our study quantitative method was used in the first phase to gather feedback from students followed by the second phase of qualitative methods in which the curriculum was reviewed for its alignment to the objectives and implementation, and discussions were conducted with teachers and students to explain the findings obtained from the quantitative survey and enrich the information gathered.

The Context, Input, Process and Product (CIPP) model was found to be most relevant to the objectives of the study as it focuses not only on whether the program is working but also helps in identifying areas of improvement. [25, 26]. This model was used to develop questions related to context (integration in curriculum), input (clarity of course contents, relevance of contents to practical issues) and process (teaching learning methods used, assessment processes, student engagement), among others. The questionnaire for the quantitative phase had questions relevant to these areas, which had to be rated on a Likert scale. The questions were reviewed by a group of evaluation experts for relevance and clarity.

The focus group discussion (FGD) guide for students focused on course contents, integration, instructional methods, transition from theory to practice and student achievement (knowledge, skills and attitude). Faculty FGD included questions around integration, student engagement,course contents methods, assessment along with challenges confronted.

### Quantitative Phase

Quantitative data was collected by an online questionnaire using SurveyMonkey. The structured questionnaire was offered to all students (500 in total) from Years 1–5 after pilot testing. Pilot was carried out on 10 students and as no changes were indicated so responses of the pilot were included in the study results. Multiple techniques were utilized to maximize the return rate. The questionnaire link was posted on a curriculum management software visited by students often to check their schedule and announcements. The link was also posted on student WhatsApp groups and Facebook pages so that they could conveniently respond in their free time. Protected time was also given following teaching/learning sessions to facilitate completion of questionnaire. Hard copies were distributed during the protected time especially for the benefit of those students who were not carrying their cell phones or laptops or could not access the online form. The data was coded to maintain anonymity and confidentiality of the participants.

### Qualitative Phase

Qualitative data was collected through document review and three focus group discussions (FGD) two with students and one with faculty. Semi-structured interview guide was used for the FGDs. Two separate FGDs were carried out with students of Years 1 & 2 and Years 3–5 who had completed the survey questionnaire and responded to the FGD invites sent by emails; their consent was taken at the start of FGD. Faculty FGD was conducted with the faculty actively involved in teaching bioethics, who were also part of Bioethics teaching group. Interviews were conducted with the clinical faculty who had either taken a session or led specific subject-related discussion on bioethics issues, for example, clinical faculty of Department of Gynecology and Obstetrics for sessions on ethical issues in reproductive health were included.

A review of the proposed revisions and the curriculum being implemented was done to determine if the objectives, contents and pedagogies were being implemented as planned. Secondary data analysis was done based on student feedback and evaluation, pre and post session meetings and faculty feedback and suggestions.

### Ethical Considerations

Ethics approval was obtained from The Aga Khan University Ethical Review Committee before initiating the study. Informed consent of participants was taken at several points. In the quantitative phase of the study, a consent form preceded the questionnaire, providing all pertinent details about the study objectives, questions, and an assurance regarding the voluntary nature of participation. Confidentiality was assured by keeping anonymity and allocating code number to each participant.

The focus group discussion participants were briefed about the study and its objectives both at the time of obtaining their informal informed consent and at the time of the focus group discussion. The process was repeated prior to the commencement of the FGD, participants being given assurance regarding confidentiality and the freedom to withdraw should they choose. In addition, they were also given an opportunity to question the investigators and clarify their understanding about the research. Also, their written informed consent was sought before initiating the audio recording process.

## Results

### Quantitative data analysis

The questionnaire return rate was 67.4% of which 31.45% responses were from Year 1 students, 19.58% from Year 2, 16.62% from Year 3, 14.54% from Year 4 and 17.80% from Year 5. students respectively.

The quantitative data was analysed for frequency of responses to the different statements on the Likert scale. Students’ perception of their achievement was assessed in terms of acquisition of knowledge and skills along with development of appropriate attitude and ethical behaviour. For the purpose of analysis agree and strongly agree were collapsed to ‘agree’ and disagree and strongly disagree were collapsed to ‘disagree’.

Majority (71%) of students agreed that the knowledge acquired from the bioethics sessions helped them identify the rights and responsibilities of patients and the rights and responsibilities of health professionals (67%). Moreover, 66% students agreed that the learning helped them identify ethical issues in the various domains of bioethics, discuss constituents of moral positions (62%) and understand the pros and cons of different moral positions (63%). More than half (61%) of the students agreed that knowledge gained from bioethics curriculum helped them relate contemporary issues in bioethics with concepts learnt and 60% endorsed that the sessions helped them understand the application of ethics in their professional life.

(Fig. 1).

**Fig. 1:**
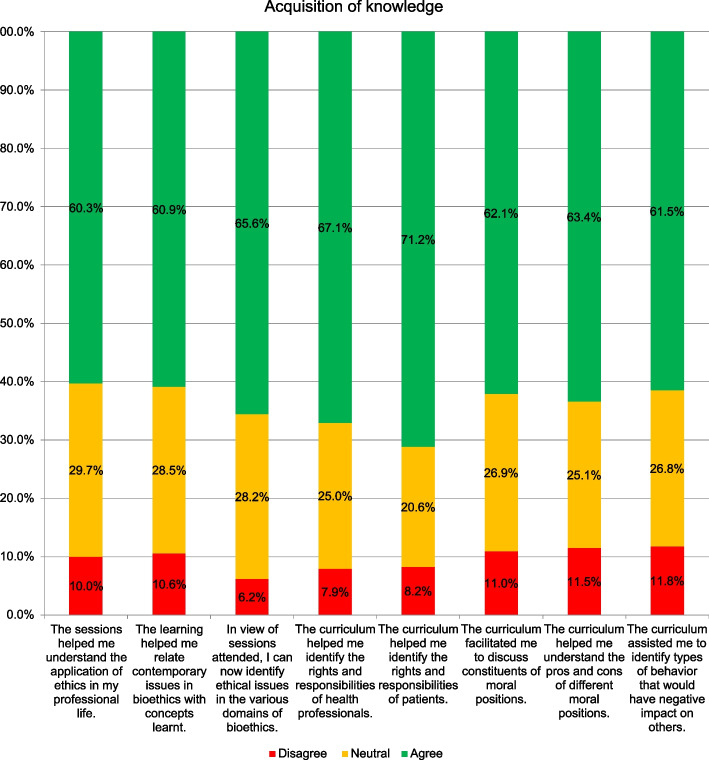
The percentage of students who agreed, were neutral, disagreed with the statements regarding their perception on their achievement in terms of acquisition of knowledge from the bioethics curriculum

Overall, more than half of the students agreed that the curriculum contributed to skill development. 60% agreed that the skills acquired helped them build a moral argument to resolve ethical dilemmas, 60% stated that the curriculum equipped them to use critical thinking skills, 59% to use reasoning skills and 59% to use active listening as tools to resolve ethical dilemmas.

(Fig. 2).

**Fig. 2:**
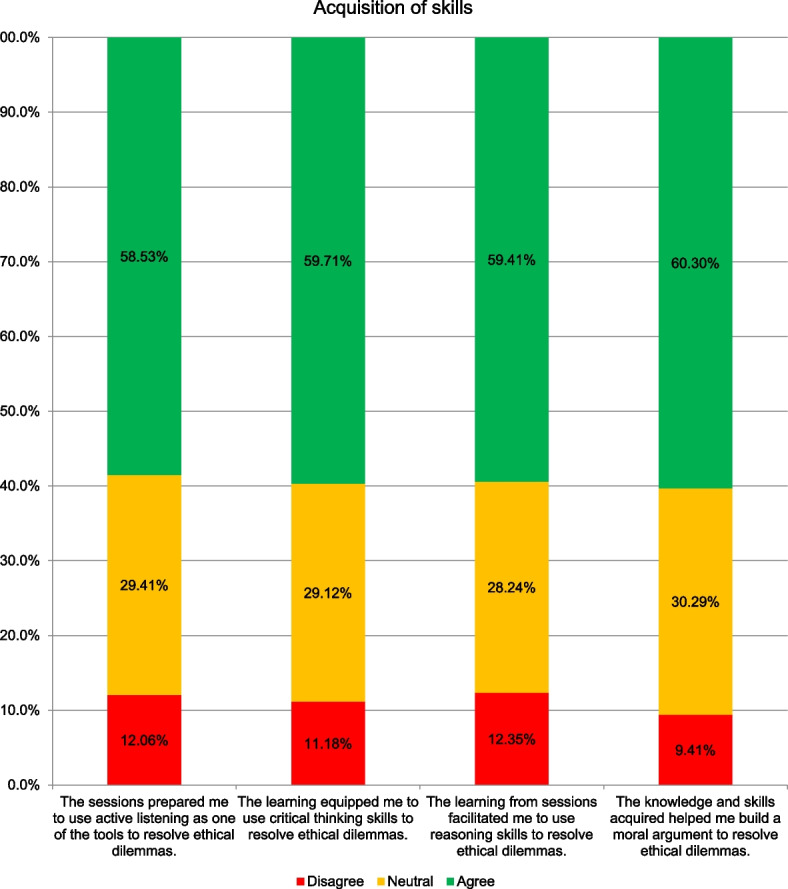
The percentage of students who agreed, were neutral, disagreed with the statements regarding their perception on their achievement in terms of development of skills from the bioethics curriculum

**Fig. 3:**
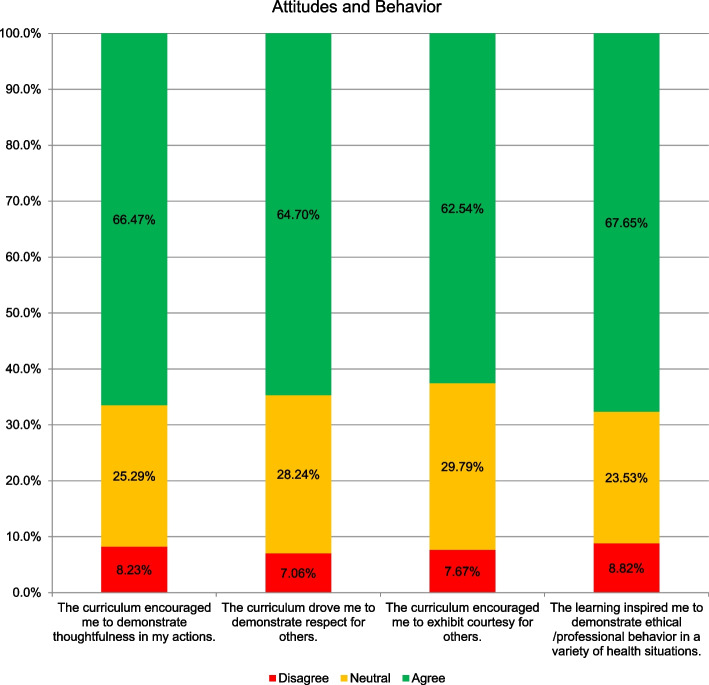
The percentage of students who agreed, were neutral, disagreed with the statements regarding perception of their achievement in terms of their development of appropriate attitude and ethical behaviour from bioethics sessions

Majority (68%) of the students opined that the learning inspired them to demonstrate ethical/professional behaviour in a variety of health situations. Moreover, 67% students agreed that the curriculum encouraged them to demonstrate thoughtfulness in their actions, while 64% and 63% agreed that the curriculum encouraged them to demonstrate respect and courtesy for others, respectively (Fig. 3).

Further quantitative data was analyzed descriptively. The data showed that students across all years strongly agreed/agreed with the statements on the questionnaire (ranging from 75—83.33%). Year 5 students had the highest percentages for agreement across almost all statements on the questionnaire followed by Year 2 students (highlighted in dark green and light green in Table 1).

**Table 1:**
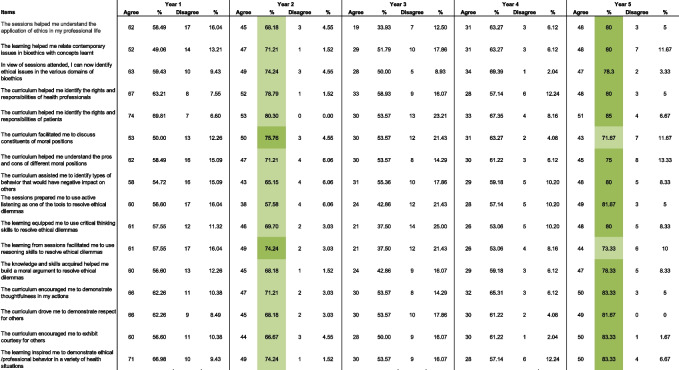
Year-wise student responses to questionnaire. Percentage of responses of students across all years (1–5) to the statements on the questionnaire regarding acquisition of knowledge, skills and attitude/behavior. Dark green indicates the highest percentage of agreement and light green second highest

### Qualitative data analysis

Group data is reported to ensure that confidentiality is maintained. For qualitative phase, data was transcribed verbatim and then independently analyzed by the three researchers. Using content analysis techniques, themes and subthemes were generated. The focus group discussion was recorded with participants' informed consent and transcribed verbatim. It was checked for accuracy by the first author. The transcribed notes were reviewed by the three authors independently who identified codes and categorized them. The codes and categories were then shared, and any differences were sorted out and consensus reached followed by identification of themes. The themes with relevant quotes are discussed in Table 3.

Based on the findings of the quantitative survey the researchers asked questions to identify specific strengths of the content and instructional methods that the students found useful or vice versa.

### Course contents

Students of clinical years commented that the contents of the bioethics curriculum were very relevant and helped them approach ethical issues seen in the clinics from multiple perspectives.

“Topics covered were very important and necessary for us as medical students. It helped us approach ethical issues from multifactorial perspectives. We see their application in our clinics today” (Year 3 student).

Another Year 3 student endorsed the same, commenting “I feel the bioethics curriculum; the fact that it is mandatory is useful because the basic principles that have been touched upon and the main theories, we have talked about proved to be very useful.”

Other comments included:

“Subtopics selected are excellent, a more clinical-oriented approach is effective, for example the sessions that I recall about renal dialysis, were very clinically oriented so we got to know the patient perspective because telling somebody to be empathetic is fundamentally useless but showing us where that empathy lies and where it should go is far more effective.” (Year 3 student).

“The curriculum helped me understand the individual patient and not only the disease! I think that it helped me become more empathetic and humane. So, at the end we are just not a clinician, not just a robot treating symptoms, we are treating the patients themselves as a whole.” (Year 4 student).

The students in Years 1 and 2 felt that learning on simulated patients was most useful. Some felt that the theory is not so important and bioethics sessions should not be mandatory throughout. Participants also suggested for more of clinically and contextually relevant scenarios to be to be used for discussion.

“The topics are very important and simulated patients are very interesting.” (Year 2 student).

“Some sessions that we have had like breaking bad news, dealing with social stigmas, perspectives of patients where we had simulated patients were very effective” (Year 3 student).

“We should have more and different cases where students can practice the foundations of bioethics, there was one case in which we had patient of Down’s syndrome, and we had a case where we had to break bad news for cancer. I think I have seen these cases twice. I would want that there be more cases and more variations. Repetitions should be avoided” (Year 2 student).

“The sessions are mandatory for us in Years 1 and 2, I feel that an option [be given] to students who really care about these things. For the first six months or a year, [there can be] mandatory bioethics sessions and after that reduce it to a CME credit course for students who optionally sign up for it.” (Year 2 student).

The FGDs revealed that understanding of importance and impact of bioethics learning was gradually realized as students progressed through the five years.

Faculty was satisfied with the course contents and the way in which the content was sequenced during the undergraduate years form theories, principles and concepts to application. However, many felt that it could be further pursued how learners translate this knowledge in their day-to-day interactions. Some comments by faculty on bioethics curriculum contents were:

“As far as the content is concerned, I think it is quite well laid out. If you see in the first year, we introduce the history of ethics and ethical principles. In the second year, the focus is more on ethical issues, and we also integrate ‘Rights’ as well and then clinical ethics. But what I noticed, as part of their skill development and attitude development, I think that part needs to be followed up. On theory, I think we are really good and introduce theories and concepts very well, but how do students translate those concepts and practice it in their day-to-day routines is important. I think that is something that I would like to pursue.” (Senior Instructor, Department of Community Health Sciences).

“I can see that one part is of introduction and key concepts, and the other part is history and principles, and the larger part is of ethical issues. So, are we trying to articulate and synthesize all of this and say to the student, look in the first year you will be working on principles and concepts and in the second year more of issues” (Senior Instructor, Department of Community Health Sciences).

“Our students and all of us have our prejudices, how we react to the poor, how we react to the women or to the very old or to the child. And somehow, I think in the curriculum we have not integrated this that on what basis do you interact with the other. It is a topic which we can easily integrate within our curriculum.” (Associate Professor, Department of Community Health Sciences).

### Instructional methods

With regards to the instructional methods, it was commented generally that interactive sessions engage the students better and enhance their learning. Multimodal instructional methods were appreciated. Small group teaching was commented to be more effective in terms of generating rich discussion. It was suggested that large class format should be limited and used only when needed. Some comments by students were as follows:

“About teaching styles…we liked that it was interactive, and we appreciate the effort.” (Year 2 student).

“It is important that faculty has taken initiative to make it interactive so we remember what we should do and what we should not do in clinical settings.” (Year 2 student).

“When they add a movie or when they add a video or when they facilitate a session where there is more interaction, like they make small groups, do role plays, those things actually help, basically clinical scenarios help. When the first years go into the third year, it actually helps.” (Year 3 student).

“I think teaching learning methods are great, but we should have smaller groups which would help a lot. The scenarios have been very effective; we have had difficult scenarios which have been taught by excellent facilitators.” (Year 5 student).

Faculty was satisfied with the pedagogies being used (movies, student presentations followed by discussions, role plays, interactive lectures with videos and worksheets, panel discussions, dialogue and discussion and case scenarios) emphasizing that student engagement and learning were to be achieved and the strategies used worked well. However, they proposed that journal writing and storytelling could also be introduced.

Some comments by faculty were:

“I try to focus on constructivist approach to teaching and learning where we use worksheets so that student engagement is there. Worksheets are more likely to make students reflect and make sense of the dilemma presented or the concept being discussed.” (Senior Instructor, Department of Community Health Sciences).

“We also tried dividing the 100 (students) into groups of 50. I found engaging the 50 was so much easier!” (Associate Professor, Department of Community Health Sciences).

Data triangulation from the quantitative phase and qualitative phase of study (including the FGDs, student feedback and evaluation, pre- and post-session meetings and faculty feedback and suggestions) further helped identify the strengths of the curriculum along with challenges confronted and suggestions for way forward. Six common themes emerged related to curriculum (course contents, mode of instruction, integration, and assessment), student engagement and achievement. The themes, their strengths, challenges and recommendations are listed in Table 2 below.

**Table 2:** Strengths and challenges related to curriculum and practices based on student and faculty FGDs. The strengths, challenges and recommendations of the common themes that emerged related to curriculum (course contents, mode of instruction, integration, and assessment), student engagement and achievement

Current curriculum/practice	Strengths	Challenges/Recommendations
Course Contents	Well sequenced Relevant topics Clinically oriented cases Basic principles and theories useful Well built up from concepts to application	Consistency in delivery by diverse facilitators (clinical/basic science faculty) More contextually relevant cases
Mode of Instruction	Interactive sessions Role plays interesting Simulated patients effective Well-made Scenarios Small group activities work well Discussions improved understanding Movies/video clippings effective resource	Teaching in small groups (multiple facilitators required for small group work, multiple venues and logistics required for small group activities) Involvement of clinical faculty
Integration	Good integration in modules seen in Years I and II	Better integration required in PBLs and PSILs PBL facilitators to be better equipped for addressing ethical issues
Assessment	Student worksheets help assess student learning Student assignments reflect their learning Student presentations show understanding Reflexive notes reflect their thoughts, feelings and bias	Introducing summative assessment
Engagement	Concurrent sessions with smaller groups facilitated discussion Interactive lectures helped keep students engaged Reflexive/reflective notes show student involvement with the subject In class worksheets improved learning and engagement Questioning increased critical thinking	Multiple faculty (2–3 faculty required for concurrent sessions) Faculty with diverse experience (faculty with a background of bioethics and clinical faculty) Multiple venues (availability of more than one venue for each session) Logistics/resources (in terms of venues, equipment)
Student achievement (In terms of personal and development-as quoted by students)	Broadened understanding and helped understand the individual case and not only the disease Helped become more empathetic and humane Contributed significantly to critical thinking, reasoning, and problem-solving skills Assisted in setting clear cut professional norms/boundaries Not only helped become a better practitioner but a better person Increased sensitivity to ethical issues Changed perception of role of health care provider Helped differentiate between right and wrong Reflection on own and others’ behaviors	Limited self-reflection ensuring skill development

Qualitative data analysis showed similarities and differences in the perceptions/opinions of students and faculty, common recurring themes were identified as in Table 3. Similarities were found in some areas such as sequencing and integration of bioethics teaching along with contextual and cultural relevance. There was a difference in opinions regarding mandatory attendance for all bioethics sessions and types of assessment. Some students of earlier years opined that sessions should not be mandatory, whereas students of later years emphasized the sessions to be mandatory. However, all faculty emphasized that they should be mandatory and remain a criterion for eligibility of module exam and end of year exam. Also, students were comfortable with the formative mode of assessment, whereas faculty suggested that there should be summative exam along with formative assessment (Table 3).

**Table 3:** Similarities and differences in the perceptions/opinions of students and faculty. Similarities and differences in the perceptions/opinions of students and faculty regarding sequencing and integration of bioethics sessions, teaching of theoretical concepts, contextual and cultural relevance, mandatory nature of sessions, mode of assessment and recommendations for the future

Theme	Students’ opinion/perception	Faculty opinion/perception
Sequencing /integration of contents/sessions	Well sequenced, further integration in the clinics is required Better integration required in PBLs and PSILs *“Topics covered were very important and necessary for us as medical students. It helped us approach issues / multifactorial issues and address them, we see their application in our clinics today”* (Year 3 student) *“Yes, situations which we were given were beneficial…but when we see such cases in clinics then we can* (better) *communicate with them.”* (Year 4 student)	Sequenced well from theories to application in clinical settings Integration required in PBLs *“I feel satisfied with the sequencing because I think when we start with Year I, that is where the larger backdrop is created. They get introduced to basic concepts and how the concepts are integrated. I even now recall this slide that all possible aspects of bioethics is noted and then we ask the students what pulls them all together? How do we integrate? And I love the responses.”* (Associate Professor, Department of Community Health Sciences)
Theoretical concepts/contents/ /clinical application	Diverse opinion. Most said basic concepts are important and lay the base for application. Few commented that history and theories were not of much use *“So yes the theory is there and it translates into performance.”* (Year 4 student)	Historical background, knowledge of concepts very relevant *“As far as the content is concerned, I think it is quite well laid out. So if you see in the first year we introduce ethics history and principles. In the second year the focus is more on ethical issues and we also integrate right as well and then clinical ethics.”* (Senior Instructor, Department of Community Health Sciences)
Contextual/Cultural /Clinical relevance	Very important All cases should be clinically/contextually relevant *“I would recommend a more clinical oriented approach, the session that I recall was about renal dialysis,it was very clinically oriented, we got to know the patient perspective because telling somebody to be empathetic is fundamentally usesless but showing us where that empathy lies and where it should go is far more effective.”* (Year 2 student)	Cultural and regional norms very important All bioethics teaching should be contextualized and culturally relevant *“It is very important that we should look at bioethics curriculum with our own cultural, regional norms and settings”.* (Senior Instructor,Pathology and Laboratory Medicine) *“Ethics is about what shouldn’t be the case and it is not only about looking and accepting what is the case. A lot of our cultural norms, we can debate and say they are unethical.”* (Associate Professor, Department of Community Health Sciences)
Mandatory nature	Diverse opinion, earlier years said it should not be mandatory while later years commented that sessions should be mandatory *“Because of the mandatory participation, people feel kind of obligated to be there, so they don’t really engage, they feel that it’s something being forced upon them.”* (Year 1 student) *“I feel the bioethics curriculum the fact that it is mandatory is useful because the basic principles that have been touched upon and the main theories, we have talked about proved to be very useful.”* (Year 3 student)	Needs to be mandatory & attending all sessions should be one of the eligibilities for appearing in end of module exam
Assessment	Should remain formative *“I think it should be as it is, now its formative.”* (Year 2 student) *If we're going to have some sort of assessment that should not be aimed to be summative.”* (Year 1 student)	Should include summative assessment along with formative *“I think summative part (assessment) is important.”* (Senior Instructor, Pathology and Laboratory Medicine)
Recommendations	Sessions should be shorter Sessions should be well spaced and not close to any exam Add the topic of ethics in relation to social media “*Sessions should not be placed right before an exam, definitely not in the exam week.”* (Year 4 student) *“…because now we live in this era where we have mobile phones and internet if we could have sessions how to maintain patient confidentiality, when we are posting whatever case we saw on social media, what are the things we should be careful about.”* (Year 4 student)	Add organizational ethics, professional ethics, and public ethics *“We tend to focus more on clinical ethics rather than on public health. I think public health, the community health perspective seems to get marginalized or missed altogether, although I believe it can be integrated with every clinical aspect also. So that bit needs strengthening.* (Associate Professor, Department of Community Health Sciences) *"Regarding content, I would suggest that we focus on professional ethics part near the third year or at the end of the second year." *(Assistant Professor, Department of Surgery)

## Discussion

This bioethics curriculum evaluation study was carried out to determine the effectiveness of the bioethics curriculum, specifically with respect to student achievement, appropriateness of course contents, and effectiveness of instructional methods used to engage the students.

Student achievement was gauged in terms of student’s perception of acquisition of knowledge and skills and development of professional behavior. Møller (2020) endorses involving students perspective stating it to be relevant for bridging the gap between theory and clinical reality [27]. The quantitative phase findings related to student achievement in terms of knowledge, skills and attitude were in line with qualitative findings wherein students attributed the contents of the bioethics curriculum contributing to student achievement in terms of ability to contribute to moral positioning and ethical decision making.

The evaluated bioethics curriculum runs longitudinally throughout the 5-year undergraduate curriculum which has been stressed by other studies too as this gives opportunities of application of knowledge and skills learnt [12, 28, 29]. Study findings showed that students valued the teaching of bioethics and its contribution to personal and professional development for ethical professional behavior and decision making more so in the later years. A study from Japan has also related bioethics and professionalism and emphasized professional ethics for medical students [30].

The qualitative component of the study found the overall curriculum content to be relevant, though the importance of this was realized and appreciated more during clerkship where students could apply or relate to the concepts learnt in earlier years. Quantitative phase was in alignment as students of Year 5 had highest percentage of agreement related to acquisition of knowledge, skills and attitude from the bioethics curriculum. Our study found the curriculum to be culturally relevant and contextual, the cases used to be relevant and contextual to both urban and rural settings and addressing ethical issues pertaining to different socioeconomic backgrounds, classes, gender, among others. Students at Aga Khan University Medical College practice globally and come from diverse settings and different provinces of the country each having its own culture and nuances, hence cultural relevance is imperative. Studies have highlighted the relevance of understanding culture and religion for a diverse group of students [13, 31, 32]. Moreover, bioethics curriculum tailored culturally has been emphasized for formalization of bioethics in the curricula of medical schools [33].

Our study findings revealed that the use of multimodal instructional methods including interactive lectures, case based discussions, brainstorming, problem solving exercises, role plays, case studies and workshops were effective in enhancing student engagement and learning. Another study emphasized the role of lecture emphasizing them to be informative helping students to change the way they see things and to establish boundaries [34], while another study shows preference for interactive sessions and minimal lecturing [35]. Gulino et al. also endorse case studies and interactive teaching methods for bioethics teaching [36], and Ashfaq et al. found case based discussions and use of mass media and popular culture useful for bioethics teaching [37]. Our study has also found that use of visual tools such as movies/medical dramas and comics to be very effective and potentially useful as teaching tools. Ike et al. also suggests that visual tools such as comics and films grasp attention, facilitate comprehension, promote recall of embedded messages and have the ability to engage young minds [38]. Cambra et al. have also found medical dramas potentially useful as teaching tools for discussing issues related to bioethics and professionalism [39]. Rameshkumar endorses interactive sessions, maximizing student interaction and use of films to be effective [40]. Miles et al. find case discussions important adding that they teach sensitivity to moral aspects of medicine, however, warn that care in selection and discussion of these cases is crucial [41]. Moreover, Sullivan et al. propose that student engagement in curricular development, reflective practice in clinical settings, and peer-assisted learning are strategies to enhance clinical ethics education [42].

D’Souza et al. [12] report that longitudinal integration of bioethics curricula in preclinical and clinical years helps overcome the translation gap. Kasulkar et al. also stress on continuing ethics education and reinforcement throughout the undergraduate years further in internship and postgraduate periods [43]. However, our study finds that longitudinal integration may minimize the gap, but students still felt a disconnect from theory to practice and attributed this gap to limited role modelling, personal bias, varying expertise of clinical faculty and time constraint.

The qualitative arm of our study showed that students’ perception regarding contribution of bioethics to their personal and professional development increased as they progressed through the program. Senior students could better appreciate the positive impact of learning on their ethical positioning and decision making. They felt knowledgeable and competent enough to holistically comprehend and apply the learnt ideas and concepts. The quantitative results endorsed the qualitative as students across all years agreed that learning from bioethics curriculum contributed to their development in terms of acquisition of knowledge, skills and attitude with Year 5 having the highest percentages for agreement across all statements on the questionnaire followed by Year 2 students. The results make it difficult to draw broad conclusions with some studies suggesting that moral reasoning ability is either not affected or negatively affected and others suggest they are affected positively [2].

It is important to consider the results presented here in the context of the private medical college where the study was conducted and the sociocultural context of Pakistan. Students enter medical college after 12 years of schooling in natural sciences at the average age of 18 years. They have no prior formal exposure to bioethics or other social science subjects such as philosophy. This is compounded by the fact that the country has weak socio-political and regulatory systems that have failed to address poverty, education and health inequities. In this context, an early and continued exposure to ethics through a longitudinally integrated curriculum is critical to develop required competencies in future healthcare practitioners. The curriculum itself requires support for effective implementation. The institution provides faculty development opportunities for student-centered pedagogical approaches and there has been a concerted effort to engage faculty possessing specialized knowledge in both bioethics and medical education through the establishment of a Bioethics Teaching group. In addition, there is a dedicated faculty coordinator for longitudinally taught content. Further, administrative and logistical support is required for smoother delivery of the curriculum. This is provided by a dedicated staff that ensures availability of adequate spaces and resources for teaching and learning.

## Conclusion

This bioethics evaluation study, shall contribute to the existing body of knowledge on the development and execution of bioethics curricula in medical colleges around the world. We believe that the findings could be used by curriculum developers and teachers far and wide to develop, expand and assess bioethics curricula in medical institutions globally as well as to set the stage for future studies to address the key challenges we have identified to further strengthen its impact on the practice of clinical medicine.

Bioethics curriculum should be longitudinally integrated through all the years of undergraduate medical education. Bioethics teaching sessions should be interactive with use of multimodal instructional methods to increase student engagement and foster opportunities for discussion and dialogue. Curriculum could be strengthened by integration in modules and clerkships, developing strong connection between theory and practice, ensuring role modelling and providing opportunities for application in various health care settings. The gap between theory and practice needs to be addressed in the clinical settings to provide continuity of learning experiences; involvement and commitment of the clinical faculty is essential.Periodic evaluation of bioethics curricula should be carried out to assess its effectiveness.

While there is room for improvement within the curriculum studied here, the lessons learnt through this evaluation can be utilized globally by other medical schools for development and improvement of bioethics curricula. Also, as the study took into account the social and cultural context of a developing country, we believe that the findings could potentially be used to develop and assess bioethics curricula in other local and regional medical institutions.

The present study was conducted over a period of one year in which students of Year 1—5 were assessed in terms of their personal and professional development linked to learning from bioethics curriculum. The cross-sectional nature of the study did not allow us to follow the same participants through their medical journey to assess how their knowledge, skills and attitude change over time.

Future studies can focus on one cohort of students and trace them for their entire five-year journey for a more longitudinal perspective and evaluation.

## Data Availability

The datasets used and/or analyzed during the current study available from the corresponding author on reasonable request.

## Abbreviations

DED: Department for Educational Development
CIPP: Context, Input, Process and Product
FGD: Focus group discussion
LMIC: Low- and middle-income countries
PBL: Problem-based learning
UGME: Undergraduate medical education
